# Comparison of surgical outcomes with and without Ologen collagen matrix augmentation during XEN gel stent implantation

**DOI:** 10.1186/s12886-022-02668-5

**Published:** 2022-11-08

**Authors:** Jimin Park, Joong Won Shin, Kyung Rim Sung

**Affiliations:** Department of Ophthalmology, Asan Medical Center, University of Ulsan College of Medicine, 88, Olympic-Ro 43-Gil, Songpa-Gu, Seoul 05505 Republic of Korea

**Keywords:** XEN, Ologen, Mitomycin C, Open-angle glaucoma

## Abstract

**Background:**

To compare the surgical outcomes and postoperative complications with and without Ologen collagen matrix augmentation during XEN gel stent implantation.

**Methods:**

We retrospectively analyzed patients who underwent XEN gel stent implantation with an ab externo technique. The amount of intraocular pressure (IOP) reduction, percentage of postoperative complications and additional management, and surgical success defined as IOP reduction greater than 20% compared with the preoperative IOP measurement were compared between Ologen-augmented and non-augmented groups. Groups of patients who underwent XEN gel stent implantation alone and combined with phacoemulsification were analyzed separately.

**Results:**

A total 103 eyes of 103 participants were included. Of those, 72 eyes underwent standalone XEN gel stent implantation: 42 eyes with Ologen augmentation (Oloxen group) and 30 eyes without Ologen augmentation (Xen group). Thirty-one eyes underwent XEN gel stent implantation with phacoemulsification: 19 eyes with Ologen augmentation (Phaco-Oloxen group) and 12 eyes without Ologen augmentation (PhacoXen group). The surgical success rate at six months postoperatively was not different between the Oloxen and Xen groups (56.4% vs 43.3%, *P* > 0.05) or between the Phaco-Oloxen group and PhacoXen group (57.9% vs 41.7%, *P* > 0.05). The prevalence of postoperative hypotony, 5-fluorouracil injections, use of anti-glaucoma medications, bleb needling, and additional glaucoma surgeries was not different between the Oloxen and Xen groups or between the Phaco-Oloxen and PhacoXen groups when assessed six months postoperatively.

**Conclusions:**

All groups showed significant IOP reduction after XEN gel stent implantation, but there was no significant difference between the Ologen collagen matrix augmented and non-augmented groups in surgical outcomes.

## Background

Glaucoma is one of the leading causes of irreversible blindness, affecting more than 70 million people worldwide [1]. Trabeculectomy is considered the standard surgical treatment to effectively lower intraocular pressure (IOP) for uncontrolled glaucoma with maximally tolerated medical therapy (MTMT). The success rate has increased with the use of antimetabolites, but safety issues such as hypotony, bleb infection, or progressive bleb fibrosis that leads to surgical failure remain [2–4].

The current developments in minimally invasive glaucoma surgical devices have given surgeons new and safe therapeutic options. The XEN® gel stent (Allergan, Dublin, Ireland) is a novel instrument that was developed to reduce IOP. Currently, the model measuring 6 mm in length with a 45-μm lumen connecting the anterior chamber and sub-conjunctival or sub-tenon spaces to maintain aqueous flow is used worldwide [5]. This new technique showed a non-inferior success rate and less surgical complications than conventional trabeculectomy; however, the number of postoperative needling procedures was increased [6].

Ologen collagen matrix (12 mm × 1 mm, ProSys International, UK) is a biodegradable material that is placed between the conjunctiva and episcleral space to prevent postoperative fibrosis [7, 8]. In addition, it acts as a reservoir by absorbing aqueous humor into its porous structure. Ologen has been used in various glaucoma surgeries recently. However, the effects of using Ologen are still controversial considering the inconsistent results. Some studies presented comparable success rate of using Ologen in trabeculectomy to mitomycin C (MMC) application [9–12], while other studies showed no significant advantages of using Ologen in trabeculectomy with MMC [13, 14]. Ologen augmentation in Ahmed glaucoma valve (AGV) implantation also showed contrary outcomes compared to conventional AGV implantation [15, 16].

There have been several studies analyzing the effects of Ologen in other glaucoma surgeries, but only one case–control study has evaluated the use of Ologen during XEN gel stent implantation [17]. Thus, in the current study, we reviewed the surgical success and complications during XEN gel stent implantation with and without the use of Ologen.

Phacoemulsification is frequently combined with XEN gel stent implantation, which showed significant IOP reduction in concurrent procedure [18]. As lens extraction itself also affects postoperative IOP measurement [19–21], several studies compared surgical success of XEN standalone and combined with phacoemulsification surgery. There was no significant difference in IOP reduction in long term follow up between two groups, although standalone surgery showed superior IOP reduction in short term follow up [22–24]. However, XEN standalone surgery revealed higher needling rate compared with XEN gel stent implantation combined with cataract surgery [24]. Considering the confounding effect of phacoemulsification in XEN gel stent implantation, we investigated the effects of Ologen augmentation separately in combined phacoemulsification with XEN gel stent implantation group.

## Methods

This retrospective study was conducted at glaucoma clinic of the Ophthalmology Department of Asan Medical Center in Seoul. All procedures performed in studies involving human participants were in accordance with the ethical standards of the institutional research committee and with the 1964 Helsinki Declaration and its later amendments or comparable ethical standards. The conduct of this retrospective study and waiver of informed consent were approved by the Ethical Committee of Institutional Review Board at Asan Medical Center (No; 2022–0951).

Patients who underwent XEN gel stent implantation with or without phacoemulsification from August 2020 to September 2021 were included in the current study. All patients underwent complete ophthalmologic examinations and following baseline data were reviewed: slit-lamp biomicroscopy, best-corrected visual acuity (BCVA), refractometry, Goldmann applanation tonometry, gonioscopy, stereoscopic optic disc/retinal nerve fiber layer (RNFL) photography, ultrasound pachymetry, axial length measurement (IOL Master 700, Carl Zeiss Meditec, Dublin, CA), standard automated perimetry (Humphrey Field analyzer with Swedish Interactive Threshold Algorithm standard 24–2 test; Carl Zeiss Meditec, Dublin, CA), and spectral domain optical coherence tomography (SD OCT) (Cirrus HD OCT, Carl Zeiss Meditec, Dublin, CA).

The inclusion criteria of this study were as follows: open-angle glaucoma (OAG), BCVA of logMAR + 0.30 (Snellen 20/40) or better, spherical refraction of –8.0 to + 3.0 diopters (D), and cylinder correction within ± 3 D. OAG was defined as an open angle on gonioscopy, retinal nerve fiber layer defects, or glaucomatous optic disc changes (neuroretinal rim thinning, disc excavation, or disc hemorrhage), and corresponding visual field defects. Participants were excluded when any ophthalmic or neurological disease other than glaucoma which can affect the optic nerve head were found. One eye was selected at random if both eyes met the inclusion criteria. XEN gel stent implantation was performed in patients with progressive glaucomatous changes that could not be controlled with MTMT and in those with an elevated IOP that could cause additional optic nerve head damage. A single experienced glaucoma specialist (KRS) performed all surgical interventions. All anti-glaucoma medications were continued up until the time of surgery.

All procedures were performed with an ab externo technique by opening the conjunctiva, as described by Panarelli et al. [25]. The procedure was performed under local (topical or sub-tenon injection) anesthesia. A 2-mm fornix-based conjunctival peritomy was made at the superocentral, superotemporal, or superonasal limbus with a blunt Wescott scissor. A 0.04% MMC sponge was applied for 2 min under the conjunctival flap, followed by irrigation. A preloaded injector with the XEN gel stent was placed 2 mm from the limbus, and the XEN gel stent was inserted externally. After insertion, the stent was repositioned so that 2–3 mm of the stent was placed in the sub-tenon space. In the Ologen augmentation surgery group, Ologen collagen matrix with a 1-cm diameter was inserted in the subtenon space, folded into a semi-circle, and placed 2–3 mm posterior to end of the XEN gel stent. The conjunctival incision was sutured with 8–0 Vicryl. In patients who underwent XEN gel stent implantation combined with phacoemulsification, conjunctival peritomy and MMC soaking were performed before phacoemulsification, and XEN gel stent implantation was performed after phacoemulsification using the same method.

The specific indications for Ologen augmentation were not determined. Ologen augmentation was performed in all surgeries performed between August 2020 and May 2021. However, beginning in May 2021, Ologen was not imported because of manufacturer and distributor issues. Thus, all procedure performed from May 2021 to September 2021 were done without Ologen.

For postoperative treatment, 0.5% moxifloxacin was administered four times per day, and 1% prednisolone acetate was administered twelve times per day and slowly tapered over 1 month. The IOP was measured at postoperative 1 day, 1 week, 3 weeks, 6 weeks, 3 months, and 6 months. Additional procedures (5-fluorouracil (FU) injections, transconjunctival bleb needling, bleb revision, and additional glaucoma surgeries) were conducted at the surgeons’ discretion. Surgical success was defined as more than 20% IOP reduction from preoperative measurement with or without anti-glaucoma medication and no additional operative interventions, such as bleb revision or additional glaucoma surgery, or persistent hypotony (IOP < 5 mmHg at ≥ 3 weeks postoperatively).

Statistical analysis was performed using IBM SPSS Statistics 22 (SPSS Inc, Chicago, IL USA). Student’s-t test, the Mann–Whitney U test, the Chi square test, or Fisher’s exact test was used for comparisons between groups. To compare the preoperative and postoperative IOP, the paired samples a t-test was used, and Student’s t-test or the Mann–Whitney U-test was used to compare IOP reduction during the same postoperative period between the Ologen-augmented augmentation non-augmented groups. *P*-values < 0.05 were considered statistically significant.

## Results

A total 103 eyes from 103 participants were included in this study. Of those, 72 eyes underwent standalone XEN gel stent implantation without phacoemulsification. Among 72 eyes, 42 eyes underwent Ologen augmentation (Oloxen group), and 30 eyes did not (Xen group). Thirty-one eyes underwent XEN gel stent implantation with phacoemulsification; 19 eyes underwent Ologen augmentation (Phaco-Oloxen group), and 12 eyes did not undergo Ologen augmentation (PhacoXen group). The baseline patient characteristics are presented in Tables 1 and 2. Ten patients had history of previous glaucoma surgery and all of them were trabeculectomy. There were no statistically significant differences in preoperative parameters between the Oloxen and Xen groups. The preoperative IOP was 17.6 ± 5.9 mmHg in the Oloxen group and 20.5 ± 6.5 mmHg in the Xen group (*P* = 0.054). The mean number of anti-glaucoma medications used preoperatively was 3.6 ± 0.8 in the Oloxen group and 3.7 ± 1.3 mmHg in the Xen group (*P* = 0.709). Similarly, there were no statistically significant differences in all parameters between the Phaco-Oloxen and PhacoXen groups. The preoperative IOP was 18.1 ± 7.0 mmHg in the Phaco-Oloxen group and 15.4 ± 5.7 mmHg in the PhacoXen group (*P* = 0.306). The mean number of anti-glaucoma medications used preoperatively was 3.6 ± 0.7 in the Phaco-Oloxen group and 3.1 ± 0.8 mmHg in the PhacoXen group (*P* = 0.101).

**Table 1:** Clinical characteristics of the Ologen-augmented (Oloxen) and non-augmented (Xen) groups during standalone XEN gel stent implantation

	**Oloxen (*n* = 42)**	**Xen (*n* = 30)**	***p*-value**
Age, years (mean ± SD)	59.81 ± 15.62	58.10 ± 15.93	0.651*
Gender, n, male:female (%)	26:16 (61.9%:38.1%)	19:11(63.3%:36.7%)	0.902^†^
Laterality, n, OD:OS (%)	17:25 (40.5%:59.5%)	16:14 (53.3%:46.7%)	0.280^†^
HTN, n, yes:no (%)	14:28 (33.3%:66.7%)	11:19 (36.7%:63.3%)	0.770^†^
DM, n, yes:no (%)	10:32 (23.8%:76.2%)	5:25 (16.7%:83.3%)	0.462^†^
Preoperative visual acuity, logMAR (mean ± SD)	0.34 ± 0.43	0.56 ± 0.51	0.059*
Preoperative intraocular pressure, mmHg (mean ± SD)	17.60 ± 5.87	20.47 ± 6.46	0.054*
Prior glaucoma surgery, n, yes:no (%)	5:37 (11.9%:88.1%)	4:26(13.3%:86.7%)	0.858^†^
Number of preoperative anti-glaucoma medications, n (mean ± SD)	3.57 ± 0.83	3.67 ± 1.32	0.709*
Preoperative diagnosis (%)			0.770^†^
Primary open-angle glaucoma	28 (66.7%)	19 (63.3%)	
Secondary open-angle glaucoma	14 (33.3%)	11 (36.7%)	
Lens, n, phakic:pseudophakic (%)	20:22 (47.6%:52.4%)	12:18 (40.0%:60.0%)	0.532^†^
CCT, (mean ± SD)	531.42 ± 46.76	535.27 ± 37.99	0.786*
ECD, (mean ± SD)	2340.36 ± 481.67	2233.23 ± 584.71	0.398*
Axial length, mm, (mean ± SD)	25.05 ± 2.19	25.91 ± 2.41	0.162*
RNFL thickness, (mean ± SD)	66.50 ± 9.86	71.56 ± 10.67	0.068*
VFI, (mean ± SD)	55.73 ± 25.11	58.89 ± 30.40	0.654*
MD, (mean ± SD)	-15.73 ± 7.90	-14.57 ± 9.15	0.590*
PSD, (mean ± SD)	9.93 ± 3.45	8.35 ± 4.75	0.154*

Abbreviations *HTN* Hypertension, *DM* Diabetic mellitus, *CCT* Central corneal thickness, *ECD* Endothelial cell density, *RNFL* Retinal nerve fiber layer, *VFI* Visual field index, *MD* Mean deviation, *PSD* Pattern standard deviation

^*^ Student’s t-test

^†^Chi-square test

**Table 2:** Clinical characteristics of the Ologen-augmented (Phaco-Oloxen) and non-augmented (PhacoXen) groups in combined Xen gel stent implantation and phacoemulsification

	**Phaco-Oloxen (*n* = 19)**	**PhacoXen (*n* = 12)**	***p*-value**
Age, years (mean ± SD)	65.53 ± 7.78	70.66 ± 7.20	0.164*
Gender, n, male:female (%)	10:9 (52.6%:47.4%)	6:6 (50.0%:50.0%)	0.886^†^
Laterality, n, OD:OS (%)	11:8 (57.9%:42.1%)	6:6 (50.0%:50.0%)	0.667^†^
HTN, n, yes:no (%)	6:13 (33.3%:66.7%)	6:6 (50.0%:50.0%)	0.452^
DM, n, yes:no (%)	3:16 (15.8%:84.2%)	3:9 (25.0%:75.0%)	0.653^
Preoperative visual acuity, logMAR (mean ± SD)	0.50 ± 0.47	0.30 ± 0.35	0.120*
Preoperative intraocular pressure, mmHg (mean ± SD)	18.05 ± 6.95	15.42 ± 5.65	0.306*
Prior glaucoma surgery, n yes:no (%)	1:17 (5.3%:94.7%)	0:12(0%:100%)	0.427^
Number of preoperative medications, n (mean ± SD)	3.57 ± 0.69	3.08 ± 0.79	0.101*
Preoperative diagnosis, n (%)			0.106^†^
Primary open-angle glaucoma	12 (63.2%)	4 (33.3%)	
Secondary open-angle glaucoma	7 (36.8%)	8 (66.7%)	
CCT, (mean ± SD)	528.84 ± 40.48	524.97 ± 42.50	0.683*
ECD, (mean ± SD)	2522.86 ± 384.39	2632.77 ± 353.25	0.367*
Axial length, mm, (mean ± SD)	23.75 ± 1.18	23.32 ± 1.69	0.646*
RNFL thickness, (mean ± SD)	65.21 ± 13.25	64.20 ± 10.51	0.872*
VFI, (mean ± SD)	40.47 ± 36.30	62.50 ± 34.46	0.243*
MD, (mean ± SD)	-20.37 ± 11.12	-13.30 ± 9.88	0.155*
PSD, (mean ± SD)	6.63 ± 3.84	6.29 ± 2.40	0.980*

Abbreviations *HTN* Hypertension, *DM* Diabetic mellitus, *CCT* Central corneal thickness, *ECD* Endothelial cell density, *RNFL* Retinal nerve fiber layer, *VFI* Visual field index, *MD* Mean deviation, *PSD* Pattern standard deviation

^*^ Mann–Whitney U-test

^†^Chi-square test ^ Fisher’s exact test

**Fig. 1:**
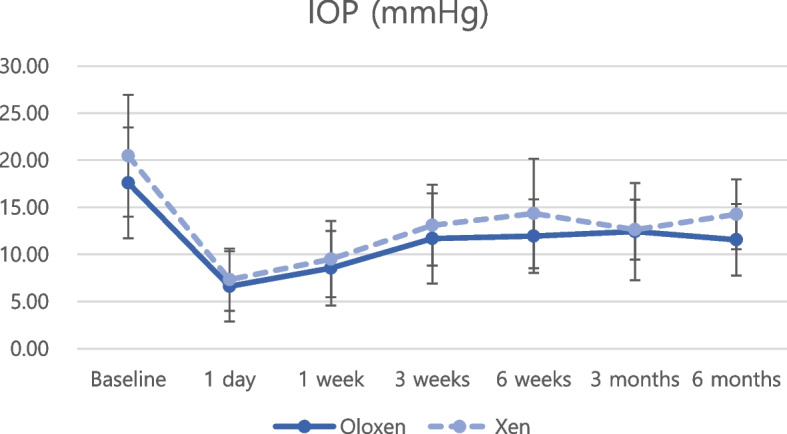
Intraocular pressure (IOP) profiles of the Ologen-augmented and non-augmented groups in standalone XEN gel stent implantation

**Fig. 2:**
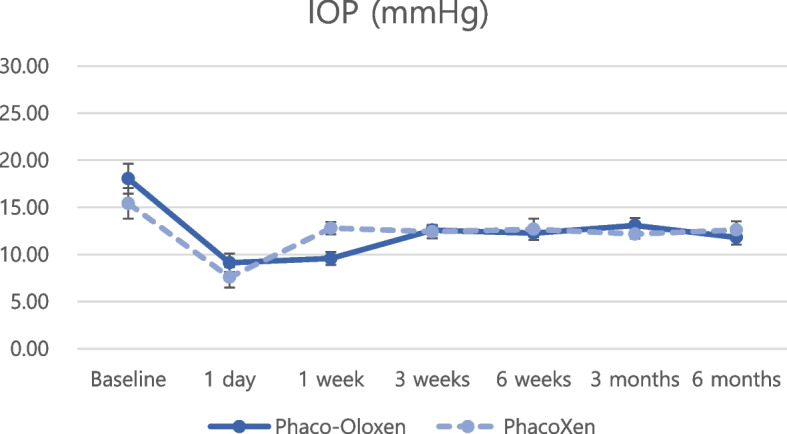
Intraocular pressure (IOP) profiles of Ologen-augmented and non-augmented groups in combined phacoemulsification and XEN gel stent implantation

**Fig. 3:**
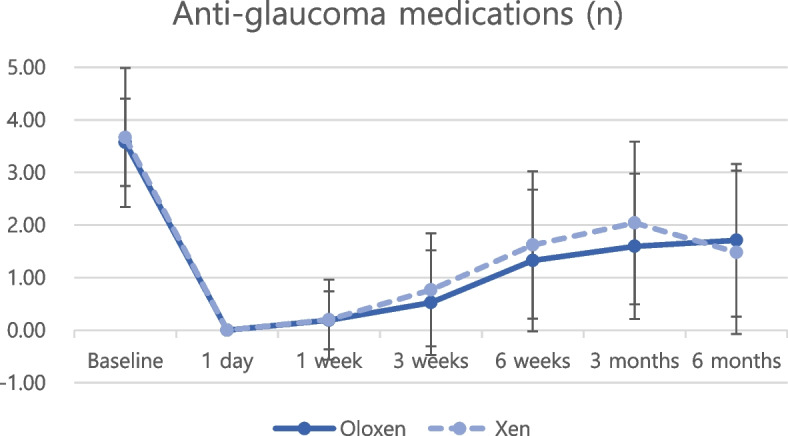
Comparison of anti glaucoma medication use between Ologen-augmented and non-augmented groups in standalone XEN gel stent implantation

**Fig.4:**
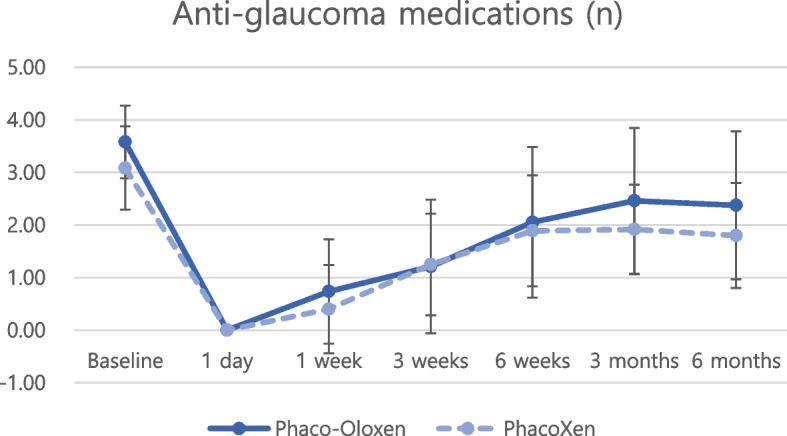
Comparison of anti glaucoma medication use between Ologen-augmented and non-augmented groups in combined phacoemulsification and XEN gel stent implantation

Regarding postoperative IOP reduction, all groups showed significant IOP reduction at every postoperative timepoint compared with the baseline IOP (Figs. 1 and 2, all *P* < 0.05 at all timepoints). Postoperative anti-glaucoma medications were also significantly reduced at every postoperative timepoint compared with preoperative anti-glaucoma medications (Figs. 3 and 4, all *P* < 0.05 at all timepoints). The amount of IOP reduction (%) was compared among subgroups (Table 3). IOP was reduced by 60.2% in the Oloxen group and by 60.9% in the Xen group on postoperative day 1. The amount of IOP reduction was decreased one week postoperatively at 48.6% and 49.9% in the Oloxen and Xen groups, respectively. At six months postoperatively, the IOP was reduced by 31.1% and 26.7% compared with the preoperative measurement in the Oloxen and Xen groups. At all-time points, the amount of IOP reduction was not different between the Oloxen and Xen groups (all, *P* > 0.05). In the phacoemulsification groups, the IOP was reduced by 46.2% and 47.8% in the Phaco-Oloxen and PhacoXen groups, respectively. Except for the first week postoperatively (*P* = 0.014), the Phaco-Oloxen and PhacoXen groups did not show significant differences (all, *P* > 0.05).

**Table 3:** Comparison of intraocular pressure (IOP) reduction (%) from baseline between the Ologen-augmented and non-augmented groups

% (mean ± SD)	**XEN standalone**			**XEN combined phacoemulsification**		
	**Oloxen**	**Xen**	***P* value**	**Phaco-Oloxen**	**PhacoXen**	***P* value**
1 day	60.3 ± 21.7	60.9 ± 20.3	0.898*	46.2 ± 26.2	47.8 ± 25.5	0.903^†^
1 week	48.6 ± 23.8	49.9 ± 23.3	0.821*	42.4 ± 21.4	11.9 ± 31.5	0.014^†^
3 weeks	28.6 ± 31.4	29.1 ± 32.6	0.941*	22.9 ± 24.7	13.4 ± 25.4	0.383^†^
6 weeks	26.6 ± 27.1 (Trabeculectomy 1)	24.6 ± 28.8	0.771*	24.4 ± 24.9 (Trabeculectomy 1)	10.3 ± 33.5	0.189^†^
3 months	27.1 ± 30.1	29.6 ± 24.6 (Trabeculectomy 1)	0.736*	23.2 ± 29.9 (Xen implantation 1)	12.9 ± 29.2	0.347^†^
6 months	31.1 ± 25.6 (Trabeculectomy 1 Ologen removal 1)	26.7 ± 20.5 (Trabeculectomy 1)	0.456*	28.7 ± 29.1	10.8 ± 32.6	0.208^†^

Parenthesis; additional surgery

^*^ Student’s t-test

^†^Mann–Whitney U-test

The surgical success rate assessed at six months postoperatively was 56.4% in the Oloxen group and 43.3% in the Xen group (*P* > 0.05). This was not different between the Phaco-Oloxen group and the PhacoXen group (57.9% vs 41.7%, *P* > 0.05).

For postoperative complications and additional procedures, the prevalence of hypotony, 5-FU injections, postoperative anti-glaucoma medications, bleb needling, and additional glaucoma surgeries were reviewed at six months postoperatively. There was no significant difference between the Oloxen and Xen groups or between the Phaco-Oloxen and PhacoXen groups (Table 4). Four cases of hypotony were found in the Oloxen group, and none were identified in the other groups. Regarding additional surgeries, five patients underwent trabeculectomy, one patient underwent additional XEN gel stent implantation, and one patient underwent Ologen removal due to migration of Ologen.

**Table 4:** Incidence of postoperative hypotony and additional procedures at six months postoperatively

	XEN standalone			XEN combined phacoemulsification		
	Oloxen	Xen	*p*-value	Phaco-Oloxen	PhacoXen	*p*-value
Hypotony	4 (9.5%)	0	0.135^	0	0	
5-FU injection	20 (47.6%)	16 (53.3%)	0.633^†^	5 (26.3%)	3 (25.0%)	1.000^†^
Postoperative medications (n)	1.72 ± 1.45	1.48 ± 1.55	0.529*	2.38 ± 1.41	2.00 ± 1.00	0.398#
Bleb needling	6 (14.3%)	2 (6.7%)	0.455^	0	0	
Additional surgery	3 (7.1%) Trabeculectomy 2 Ologen removal 1	2 (6.7%) Trabeculectomy 2	0.938^	2 (10.5%) Trabeculectomy 1 XEN implantation 1	0	0.510^†^

^*^ Student’s t-test

^†^Chi-square test ^Fisher’s exact test

^#^ Mann–Whitney U-test

## Discussion

XEN gel stent implantation has become an important surgical modality to lower IOP for patients with glaucoma. It has shown a similar success rate to trabeculectomy in previous studies [6, 26, 27]. In terms of surgical complications, XEN gel stent implantation has shown a higher incidence of postoperative bleb interventions but a lower incidence of hypotony than trabeculectomy[6]. Ologen has been utilized as a strategy to reduce bleb fibrosis in other glaucoma filtering surgeries such as trabeculectomy or AGV implantation [7, 8]. Therefore, we analyzed and compared the amount of IOP reduction and prevalence of postoperative complications and additional procedures after XEN gel stent implantation with and without the use of Ologen.

All groups showed effective IOP reduction after surgery compared with preoperative measurements, and this outcome was similar to those of previous studies, demonstrating that XEN gel stent implantation was effective for reducing IOP and the number of anti-glaucoma medications [28, 29]. On the other hand, there was no significant difference in terms of postoperative IOP reduction between the Ologen-augmented and non-augmented groups at most of the time points. The combined XEN gel stent implantation and phacoemulsification groups also showed similar results.

Aggressive wound healing with fibrosis is one of the most common reasons for surgical failure of glaucoma filtering surgery and can lead to obstruction of aqueous flow and IOP elevation [30]. Various approaches have been attempted to solve this issue [31]. Ologen, as a spacer, was developed as a solution to mechanically prevent adhesions of the conjunctiva and episcleral space. However, the effect of Ologen addition was not significant in our current study. The reason for this result might be due to the concurrent use of MMC, which is an antimetabolic agent that prevents fibrosis by inhibiting the synthesis of collagen by fibroblasts [32]. In our study we applied a 0.04% MMC sponge for 2 min in all cases. The use of this dosage of MMC has been shown to be effective for decreasing fibrosis [33], and we may speculate that the use of Ologen did not enhance the ability to reduce postoperative fibrosis when used additively with MMC application. A similar trend was found in one case–control study of XEN gel stent implantation using Ologen with MMC [17] as well as with Preserflo [34] and trabeculectomy [35]. Therefore, we speculate that Ologen does not enhance the antifibrotic effect when combined with MMC, although Ologen showed that it can replace MMC in other studies [11, 31, 36].

In terms of postoperative hypotony and additional procedures, there was no significant difference between the Ologen-augmented and non-augmented groups. This result was different from those of a previous study[17] that showed less bleb fibrosis, 5-FU injections, needling procedures in an Oloxen group than in a Xen group. This difference might be due to the difference of surgical method; we performed an open conjunctiva-ab externo technique in all cases. In the previous study, those in the Oloxen group underwent a 2.5-mm conjunctival dissection to insert Ologen, while those in the Xen group underwent surgery using a conventional technique (i.e., ab interno technique since XEN implantation was first introduced using closed conjunctival approach) [37]. One study showed a comparable success rate and a lower needling rate in an open conjunctiva group than in a closed conjunctiva group [38]. As patients in both the Oloxen and Xen groups underwent surgery using the same ab externo technique, we believe our results are more unbiased.

There were four cases of hypotony, and these were all observed in the Oloxen-augmented group. Early postoperative hypotony has frequently been observed in patients undergoing trabeculectomy with Ologen, which may be explained by the higher flow facilitated by the presence of Ologen collagen matrix [14]. XEN gel stent implantation has been highlighted as a new technique with a lower rate of hypotony than trabeculectomy due to the small lumen [18]. Approximately 6–8 mmHg of back pressure is theoretically employed to prevent hypotony. Although XEN gel stent implantation is known to be associated with less hypotony, our study showed a few cases when using Ologen. There was no hypotony maculopathy in all 4 cases, but this event still suggests that surgeons should consider hypotony when using Ologen during XEN gel stent implantation.

Regarding the prevalence of postoperative additional procedures, the use or lack of use of Ologen did not differ. However, there was one case in which Ologen needed to be removed because it migrated, which caused irritation to the patient. The patient showed good IOP control after surgery, but there was discomfort due to the migration of Ologen to the limbus; thus, it was removed six months postoperatively.

Some limitations of this study were the small sample size in each group and the short-term follow-up period of six months. These constraints present the need for a prospective, large-scale study with a longer follow-up period. The variability of the glaucoma diagnosis and inclusion of those patients with the history of prior glaucoma surgery could have also affected the surgical outcomes although previous studies showed effective IOP reduction in XEN gel stent implantation after failed trabeculectomy [39–41]. In our study, we used an open conjunctiva, ab externo approach for XEN gel stent implantation; however, XEN was originally developed for use with an ab interno approach; thus, our results should be interpreted with caution in this regard. Finally, we did not randomize the use of Ologen, which may have biased the results. However, as we mentioned in the method section, Ologen was not available due to manufacture and distributor issues during some periods; thus, we used Ologen in all cases when available and did not use it when it was not available, and the preoperative parameters did not differ between the Ologen-augmented and non-augmented groups. Further studies using other surgical methods and randomization are warranted.

## Conclusion

Our study outcome conclusively indicated that there was no significant difference in terms of IOP reduction or surgical complications between Ologen augmentation and no augmentation during XEN gel stent implantation. A similar tendency was also found when XEN gel stent implantation was combined with phacoemulsification.

## Data Availability

The data used to support the findings of this study are included in the article and are available on request from the corresponding author.

## Abbreviations

IOP: Intraocular pressure
MTMT: Maximally tolerated medical therapy
MMC: Mitomycin C
AGV: Ahmed glaucoma valve
BCVA: Best-corrected visual acuity
RNFL: Retinal nerve fiber layer
OAG: Open-angle glaucoma
FU: Fluorouracil

## References

[1] Quigley HA, Broman AT (2006). The number of people with glaucoma worldwide in 2010 and 2020. Br J Ophthalmol.

[2] Kirwan JF, Lockwood AJ, Shah P, Macleod A, Broadway DC, King AJ (2013). Trabeculectomy in the 21st century: a multicenter analysis. Ophthalmology.

[3] Edmunds B, Thompson JR, Salmon JF, Wormald RP (2002). The National Survey of Trabeculectomy. III Early and late complications Eye (Lond).

[4] Gedde SJ, Herndon LW, Brandt JD, Budenz DL, Feuer WJ, Schiffman JC (2012). Postoperative complications in the Tube Versus Trabeculectomy (TVT) study during five years of follow-up. Am J Ophthalmol.

[5] Widder RA, Dietlein TS, Dinslage S, Kuhnrich P, Rennings C, Rossler G (2018). The XEN45 Gel Stent as a minimally invasive procedure in glaucoma surgery: success rates, risk profile, and rates of re-surgery after 261 surgeries. Graef Arch Clin Exp.

[6] Theilig T, Rehak M, Busch C, Bormann C, Schargus M, Unterlauft JD (2020). Comparing the efficacy of trabeculectomy and XEN gel microstent implantation for the treatment of primary open-angle glaucoma: a retrospective monocentric comparative cohort study. Sci Rep-Uk.

[7] Chen HSL, Ritch R, Krupin T, Hsu WC (2006). Control of filtering bleb structure through tissue bioengineering: An animal model. Invest Ophth Vis Sci.

[8] Hsu WC, Ritch R, Krupin T, Chen HSL (2008). Tissue bioengineering for surgical bleb defects: an animal study. Graef Arch Clin Exp.

[9] Cillino S, Di Pace F, Cillino G, Casuccio A (2011). Biodegradable collagen matrix implant vs mitomycin-C as an adjuvant in trabeculectomy: a 24-month, randomized clinical trial. Eye (Lond).

[10] Yuan F, Li L, Chen XP, Yan X, Wang LY. Biodegradable 3D-Porous Collagen Matrix (Ologen) Compared with Mitomycin C for Treatment of Primary Open-Angle Glaucoma: Results at 5 Years. J Ophthalmol. 2015;2015(637537):1-7. 10.1155/2015/637537 PMC4452460 26078875

[11] Senthil S, Rao HL, Babu JG, Mandal AK, Garudadri CS (2013). Comparison of outcomes of trabeculectomy with mitomycin C vs. ologen implant in primary glaucoma. Indian J Ophthalmol.

[12] Perez CI, Mellado F, Jones A, Colvin R (2017). Trabeculectomy Combined With Collagen Matrix Implant (Ologen). J Glaucoma.

[13] Ji QS, Qi B, Liu L, Guo XL, Zhong JX (2015). Efficacy and Safety of Ologen Implant Versus Mitomycin C in Primary Trabeculectomy: A Meta-analysis of Randomized Clinical Trials. J Glaucoma.

[14] Rosentreter A, Schild AM, Jordan JF, Krieglstein GK, Dietlein TS (2010). A prospective randomised trial of trabeculectomy using mitomycin C vs an ologen implant in open angle glaucoma. Eye.

[15] Kim TJ, Kang S, Jeoung JW, Kim YK, Park KH (2018). Comparison of 1-year outcomes after Ahmed glaucoma valve implantation with and without Ologen adjuvant. BMC Ophthalmol.

[16] Sastre-Ibanez M, Cabarga C, Canut MI, Perez-Bartolome F, Urcelay-Segura JL, Cordero-Ros R (2019). Efficacy of Ologen matrix implant in Ahmed Glaucoma Valve Implantation. Sci Rep.

[17] Navero-Rodriguez JM, Espinosa-Barberi G, Morilla-Grasa A, Anton A (2020). Efficacy of the Ologen collagen matrix in combination with the XEN gel stent implantation in the treatment of open-angle glaucoma: A case-control study. Clin Exp Ophthalmol.

[18] Mansouri K, Guidotti J, Rao HL, Ouabas A, D'Alessandro E, Roy S (2018). Prospective Evaluation of Standalone XEN Gel Implant and Combined Phacoemulsification-XEN Gel Implant Surgery: 1-Year Results. J Glaucoma.

[19] Poley BJ, Lindstrom RL, Samuelson TW, Schulze R (2009). Intraocular pressure reduction after phacoemulsification with intraocular lens implantation in glaucomatous and nonglaucomatous eyes: evaluation of a causal relationship between the natural lens and open-angle glaucoma. J Cataract Refract Surg.

[20] Shingleton BJ, Pasternack JJ, Hung JW, O'Donoghue MW (2006). Three and five year changes in intraocular pressures after clear corneal phacoemulsification in open angle glaucoma patients, glaucoma suspects, and normal patients. J Glaucoma.

[21] Armstrong JJ, Wasiuta T, Kiatos E, Malvankar-Mehta M, Hutnik CML (2017). The Effects of Phacoemulsification on Intraocular Pressure and Topical Medication Use in Patients With Glaucoma: A Systematic Review and Meta-analysis of 3-Year Data. J Glaucoma.

[22] Fea AM, Bron AM, Economou MA, Laffi G, Martini E, Figus M (2020). European study of the efficacy of a cross-linked gel stent for the treatment of glaucoma. J Cataract Refract Surg.

[23] Lim SY, Betzler BK, Yip LWL, Dorairaj S, Ang BCH (2021). Standalone XEN45 Gel Stent implantation versus combined XEN45-phacoemulsification in the treatment of open angle glaucoma-a systematic review and meta-analysis. Graefes Arch Clin Exp Ophthalmol.

[24] Yang X, Zhao Y, Zhong Y, Duan X (2022). The efficacy of XEN gel stent implantation in glaucoma: a systematic review and meta-analysis. BMC Ophthalmol.

[25] Panarelli JF, Yan DB, Francis B, Craven ER (2020). XEN Gel Stent Open Conjunctiva Technique: A Practical Approach Paper. Adv Ther.

[26] Parra MTM, Lopez JAS, Grau NSL, Ceausescu AM, Santonja JJP (2019). XEN implant device versus trabeculectomy, either alone or in combination with phacoemulsification, in open-angle glaucoma patients. Graef Arch Clin Exp.

[27] Wagner FM, Schuster AKG, Emmerich J, Chronopoulos P, Hoffmann EM. Efficacy and safety of XEN (R)-Implantation vs. trabeculectomy: Data of a "real-world" setting. Plos One. 2020;15(4):e0231614 1-10. 10.1371/journal.pone.0231614 PMC7170231 32310972

[28] Buffault J, Graber M, Bensmail D, Bluwol E, Jeanteur MN, Abitbol O, et al. Efficacy and safety at 6 months of the XEN implant for the management of open angle glaucoma. Sci Rep-Uk. 2020;10(1):4527 1-7. 10.1038/s41598-020-61319-1 PMC7066242 32161332

[29] Buffault J, Baudouin C, Labbe A (2019). XEN((R)) Gel Stent for management of chronic open angle glaucoma: A review of the literature. J Fr Ophtalmol.

[30] Jampel HD, Solus JF, Tracey PA, Gilbert DL, Loyd TL, Jefferys JL (2012). Outcomes and bleb-related complications of trabeculectomy. Ophthalmology.

[31] He M, Wang W, Zhang X, Huang W (2014). Ologen implant versus mitomycin C for trabeculectomy: a systematic review and meta-analysis. PLoS ONE.

[32] Hollo G (2012). Wound healing and glaucoma surgery: modulating the scarring process with conventional antimetabolites and new molecules. Dev Ophthalmol.

[33] Almobarak FA, Alharbi AH, Morales J, Aljadaan I (2018). The influence of mitomycin C concentration on the outcome of trabeculectomy in uveitic glaucoma. Int Ophthalmol.

[34] Vastardis I, Fili S, Perdikakis G, Kontopoulou K, Balidis M, Gatzioufas Z (2021). Preliminary results of Preserflo Microshunt versus Preserflo Microshunt and Ologen implantation. Eye Vision.

[35] Castejon MA, Teus MA, Bolivar G, Paz-Moreno-Arrones J, Castano B (2018). Outcomes of Trabeculectomy and Phacotrabeculectomy With Collagen Matrix Implant (Ologen) and Low-dose Mitomycin C: 2-Year Follow-up. J Glaucoma.

[36] Wlaz A, Wilkos-Kuc A, Rozegnal-Madej A, Zarnowski T (2019). Phacotrabeculectomy using collagen matrix implant (Ologen (R)) versus mitomycin C: a prospective randomized controlled trial. Acta Ophthalmol.

[37] Sharpe R, Pham G, Chang P (2020). Comparison of Ab Interno XEN Gelatin Stent vs Trabeculectomy with Mitomycin C: A Retrospective Study. J Curr Glaucoma Pract.

[38] Do A, McGlumphy E, Shukla A, Dangda S, Schuman JS, Boland MV (2021). Comparison of Clinical Outcomes with Open Versus Closed Conjunctiva Implantation of the XEN45 Gel Stent. Ophthalmol Glaucoma.

[39] Karimi A, Hopes M, Martin KR, Lindfield D (2018). Efficacy and Safety of the Ab-interno Xen Gel Stent After Failed Trabeculectomy. J Glaucoma.

[40] Bormann C, Schmidt M, Busch C, Rehak M, Scharenberg CT, Unterlauft JD (2022). Implantation of XEN After Failed Trabeculectomy: an Efficient Therapy?. Klin Monbl Augenheilkd.

[41] Duzgun E, Olgun A, Karapapak M, Alkan AA, Ustaoglu M (2021). Outcomes of XEN Gel Stent Implantation in the Inferonasal Quadrant after Failed Trabeculectomy. J Curr Glaucoma Pract.

